# Sedentary Behavior Interacts with Alzheimer's Disease Biomarkers on Cognition in Older Adults

**DOI:** 10.1002/alz70860_106500

**Published:** 2025-12-23

**Authors:** Marissa A. Gogniat, Lindsey Jorda, Joseph Storey, Bindu Chunduri, Amaya Aranda, Ann D Cohen, Beth E. Snitz

**Affiliations:** ^1^ University of Pittsburgh Alzheimer's Disease Research Center, Pittsburgh, PA, USA; ^2^ University of Pittsburgh School of Medicine, Pittsburgh, PA, USA; ^3^ University of Pittsburgh Alzheimer's Disease Research Center (ADRC), Pittsburgh, PA, USA

## Abstract

**Background:**

Sedentary behavior is a highly prevalent behavior in older adulthood that is related to many poor health outcomes, including an increased risk of dementia. Given the negative impact of sedentary behavior on physical health, there has been recent interest in examining its impact on cognitive health in older adulthood. However, the association between sedentary behavior and cognition has been somewhat mixed, and the neurobiological mechanisms that may underlie these associations are poorly understood. We sought to examine how sedentary behavior may interact with Alzheimer's Disease (AD) biomarkers on cognition in older adults without dementia.

**Method:**

A subset of University of Pittsburgh Alzheimer's Disease Research Center (ADRC) Clinical Core participants with at least one week of accelerometry data and positron emission tomography (PET) imaging were included (*N* = 41, age=75±8 years, 16±3years of education, 51% Male, 90% White). Participants wore the Fitbit Inspire 3 device to measure sedentary behavior, and underwent neuropsychological testing (Uniform Data Set), and PET Imaging (global amyloid beta (Aβ), tau in the meta‐temporal region). The interaction between sedentary behavior and AD biomarkers was related to neuropsychological outcomes using a moderation model that adjusted for age, sex, race, and education. Exploratory models were repeated stratifying by AD biomarker level to examine directionality of effects.

**Result:**

Sedentary behavior interacted with global amyloid burden on letter fluency (β=‐0.05, *p* = 0.05), a language composite, (β=‐0.03, *p* = 0.04), and complex attention (β=‐0.004, *p* = 0.003). Sedentary behavior interacted with tau burden in meta‐temporal regions on confrontation naming (β=‐0.04, *p* = 0.02). When these significant interactions were probed, more sedentary time was associated with better cognition in those with low Aβ and tau burden; in those with high Aβ and tau burden, less sedentary time was associated with better cognition.

**Conclusion:**

In a sample of older adults, sedentary behavior interacted with AD biomarkers on cognition cross‐sectionally. Interestingly, the AD biomarker burden level was important in understanding the directionality of the associations between sedentary behavior and cognitive performance. This study is consistent with the importance of modifying sedentary behavior, particularly in those with elevated biomarker risk for AD.

